# RAD18 Activates the G2/M Checkpoint through DNA Damage Signaling to Maintain Genome Integrity after Ionizing Radiation Exposure

**DOI:** 10.1371/journal.pone.0117845

**Published:** 2015-02-12

**Authors:** Megumi Sasatani, Yanbin Xu, Hidehiko Kawai, Lili Cao, Satoshi Tateishi, Tsutomu Shimura, Jianxiang Li, Daisuke Iizuka, Asao Noda, Kanya Hamasaki, Yoichiro Kusunoki, Kenji Kamiya

**Affiliations:** 1 Department of Experimental Oncology, Research Institute for Radiation Biology and Medicine, Hiroshima University, 1–2–3 Kasumi, Minami-ku, Hiroshima, 734–8553, Japan; 2 Department of Molecular Radiobiology, Research Institute for Radiation Biology and Medicine, Hiroshima University, 1–2–3 Kasumi, Minami-ku, Hiroshima, 734–8553, Japan; 3 Institute of Molecular Embryology and Genetics (IMEG), Kumamoto University, 2–2–1, Honjo, Kumamoto, 860–0811, Japan; 4 Department of Environmental Health, National Institute of Public Health, 2–3–6, Minami, Wako, Saitama, 351–0197, Japan; 5 Department of Genetics, Radiation Effects Research Foundation, 5–2, hijiyamako-en, Minami-ku, Hiroshima, 732–0815, Japan; 6 Department of Radiobiology/Molecular Epidemiology, Radiation Effects Research Foundation, 5–2, hijiyamako-en, Minami-ku, Hiroshima, 732–0815, Japan; Department of Genetics and Complex Diseases, UNITED STATES

## Abstract

The ubiquitin ligase RAD18 is involved in post replication repair pathways via its recruitment to stalled replication forks, and its role in the ubiquitylation of proliferating cell nuclear antigen (PCNA). Recently, it has been reported that RAD18 is also recruited to DNA double strand break (DSB) sites, where it plays novel functions in the DNA damage response induced by ionizing radiation (IR). This new role is independent of PCNA ubiquitylation, but little is known about how RAD18 functions after IR exposure. Here, we describe a role for RAD18 in the IR-induced DNA damage signaling pathway at G2/M phase in the cell cycle. Depleting cells of RAD18 reduced the recruitment of the DNA damage signaling factors ATM, γH2AX, and 53BP1 to foci in cells at the G2/M phase after IR exposure, and attenuated activation of the G2/M checkpoint. Furthermore, depletion of RAD18 increased micronuclei formation and cell death following IR exposure, both *in vitro* and *in vivo*. Our data suggest that RAD18 can function as a mediator for DNA damage response signals to activate the G2/M checkpoint in order to maintain genome integrity and cell survival after IR exposure.

## Introduction

The RING-type (E3) ubiquitin ligase RAD18 is a key player involved in post-replication repair (PRR) that regulates ubiquitylation of proliferating cell nuclear antigen (PCNA) in response to replication stresses.[1–6] RAD18 is recruited to replication forks after UV exposure.[6–8] Ubiquitylation of PCNA mediated by RAD18 facilitates the recruitment of DNA repair factors to the sites of DNA lesions at stalled replication forks for efficient repair.[7–9] Cells deficient for Rad18 exhibit increased sensitivity to various DNA damaging agents such as ultra-violet (UV) and ionizing radiation (IR) and display enhanced genome instability.[10–15]

Mammalian cells use two major DNA repair systems, non-homologous end joining (NHEJ) and homologous recombination (HR), which are employed to repair DNA damage depending on phases of the cell cycle.[16] During the late S phase and the G2 phase, HR, which requires a sister chromatid or homologous chromosome to use as the repair template, is the predominant repair pathway. Recently, it has been reported that RAD18 also plays a role in the repair of DNA double-strand breaks (DSBs) in a manner that is independent of PCNA ubiquitylation.[9,12,13,16–20] In the G1 phase, RAD18 interacts with 53BP1 to promote DSB repair via the NHEJ pathway.[9] RAD18 is known to interact with RAD51C in the S phase after exposure to IR and this interaction may enhance DNA repair via HR. [17] However, little is known about roles of RAD18 during the G2/M phase after IR exposure. Furthermore, RAD18 has a dynamic, spontaneous localization pattern during the cell cycle, which is similar to DSB repair-associated proteins but not to PCNA.[18] It is reported that RAD18 localizes to DSBs during all phases of the cell cycle, suggesting that RAD18 has individual functions at the G1, S, G2 and M phases.[18] However, the putative role of RAD18 in response to DSBs at each phase of the cell cycle phase remains elusive.

In this study, we describe a new role for RAD18 in radioresponse of cells in the G2/M phase after IR exposure. RAD18 facilitates the DNA damage signaling pathway to activate the G2/M checkpoint. Rad18-depleted cells showed increased sensitivity to IR, in term of micronuclei formation and apoptosis after irradiation; similar IR sensitivity was observed *in vivo*. These data suggest a crucial role for RAD18 in the DNA damage response induced by IR.

## Materials and Methods

### Cell culture and RNA interference

HT1080, HeLa, HEK293 and H1299 human cancer cell lines were cultured in alpha Eagle’s medium (Sigma) supplemented with 10% fetal bovine serum (FBS) and streptomycin sulfate and penicillin. Transfections with siRNA at a final concentration of 100nM were performed using 0.5% lipofectamine RNAiMAX reagent (Life Technologies). Cells were transfected with a non-targeted siRNA negative control (si-ctrl) or RAD18 siRNA (si-RAD18), both purchased from Life Technologies. Transfected cells were cultured for 48 hrs before being used in individual experiments.

### Western blotting

Cells were separated into soluble and insoluble fractions using the complete Lysis-M, EDTA-free reagent (Roche). Proteins prepared from the insoluble fractions were electrophoresed on 5–20% sodium dodecyl sulfate-polyacrylamide gels (Atto), transferred to nitrocellulose membranes (Bio-Rad) and analyzed by immunoblotting with the indicated antibodies (Abs). Primary Abs used recognized ATM phosphorylated at Ser1981 (Rockland); p53 phosphorylated at Ser15 (Cell Signaling); H2AX phosphorylated at Ser139 (Merck Millipore); histone H3 phosphorylated at Ser10 (Merck Millipore); histone H1 (Santa Cruz Biotechnology). The rabbit polyclonal Abs against human and mouse RAD18 are described elsewhere.[4] The intensity of the band on western blotting was measured using NIH image J.

### Cell cycle analysis

For cell cycle distribution analyses, HT1080 cells were transfected with si-ctrl or si-RAD18. After 48 hrs, the cells were exposed to IR or UV and cultured for 0, 6, 12 and 18 hrs. The cells were then fixed with 70% ethanol at-20°C. The fixed cells were washed twice with PBS and then treated with propidium iodide (PI) (BD Biosciences) for 30min at room temperature and analyzed using a FACSCanto **II** (BD Biosciences). The percentage of each cell cycle phase was measured using the FLOW JO software.

### Foci formation assay using the IN Cell Analyzer

Cells were plated into 96-well plates (Ibidi). Cells in the S phase were labeled using the Clik-It EdU Alexa Fluor 488 imaging kit (Life Technologies). Cells were washed twice with PBS then treated with hypotonic buffer (10 mM Tris-HCl, pH 7.4, 2.5 mM MgCl_2_, 1 mM phenylmethylsulfonyl fluoride, and 0.5% NP40) for eight min on ice. Cells were fixed with 4% paraformaldehyde in PBS for 15 min, and then treated with 0.5% Triton X-100 in PBS for 30min. Cells were stained with a specific primary Ab, then stained with species-specific Cy3 (Jackson ImmunoResearch Laboratories), Alexa Flour 488 (Life Technologies) or Alexa 647 (Life Technologies) secondary Abs. Nuclei were labeled with Hoechst 33258. Fluorescence images were obtained using an automated fluorescence microscope and IN Cell Analyzer 2000 (GE Healthcare BioScience). Cell cycle distribution was determined by the intensity of Hoechst 33258 and EdU labeling. The number of foci per cell at each cell cycle phase were determined using the image-analysis software provided with the IN Cell Developer (GE Healthcare BioScience).

### Fluorescence intensity of γH2AX

Irradiated cells were fixed in 70% ethanol at-20°C. Fixed cells were washed with PBS and then treated with permeabilization buffer (0.5% Triton-X, 1% PBS and 0.01% NaN_3_). Following incubation with an anti-γH2AX antibody for 20 min at room temperature, a mouse-specific Alexa Flour 488 secondary Ab (Life Technologies) was added to the cell pellet. The fluorescence level of γH2AX was analyzed by using the FACS Canto II and γH2AX levels induced by IR were determined by comparing the relative mean γH2AX fluorescence intensities between non-irradiated and irradiated cells.

### *In vitro* Micronucleus assay using the IN Cell Analyzer

Irradiated cells were fixed with methanol at-20°C. Nuclei and cytoplasm were stained with Hoechst 33258 and the SYTO RNA Select green fluorescent Cell Stain (Life Technologies) respectively. The numbers of micronuclei were determined using the IN Cell Analyzer 2000. Quantitative analyses of the frequency of micronuclei were performed using the IN Cell Developer.

### Mice

*Rad18* ^-/-^ mice were generated as previously described and the crosses and genotyping of experimental mice were carried out as previously described.[10,15] This study was carried out in accordance with the recommendations in the Guide for the Care and Use of Laboratory Animals of the Hiroshima University Animal Research Committee. The protocol was approved by the Committee on the Ethics of Animal Experiments of the Hiroshima University (Permit Number: B10–23–2) All mice were maintained according to the guidelines of the Institute of Laboratory Animal Science of Hiroshima University, and all efforts were made to minimize suffering.

### *In vivo* Micronucleus assay using flow cytometry

Peripheral blood was withdrawn from the tail vein in each experimental group at 0, 24 and 48 hrs after IR exposure. Blood samples (20 μl) were analyzed using the MicroFlow^PLUS^ kit (mouse) (BD biosciences), according to the manufacturer’s instructions. More than 20,000 reticulocytes were analyzed to determine MN frequencies using the FACS Canto II.

### *In vivo* Apoptosis assay using flow cytometry

Thymocytes were isolated from each experimental group at 0, 3, 6, 9 and 12 hrs after IR exposure. The distributions of apoptotic thymocytes were then identified using a PE Annexin V Apoptosis Detection kit I (BD Biosciences). More than 10,000 thymocytes per mouse were analyzed to determine the frequency of apoptosis using the FACS Canto II. All results are presented as the percentage of apoptotic cells per sample.

### Statistical analysis

Error bar represent standard deviations. Student *t* test was used for statistical analysis. Chi-squared test was used for cell cycle distribution. Single and double asterisks indicate significant differences as p<0.05 and p<0.01.

## Results

### RAD18 was required for G2/M checkpoint activation after IR exposure

**Fig 1:**
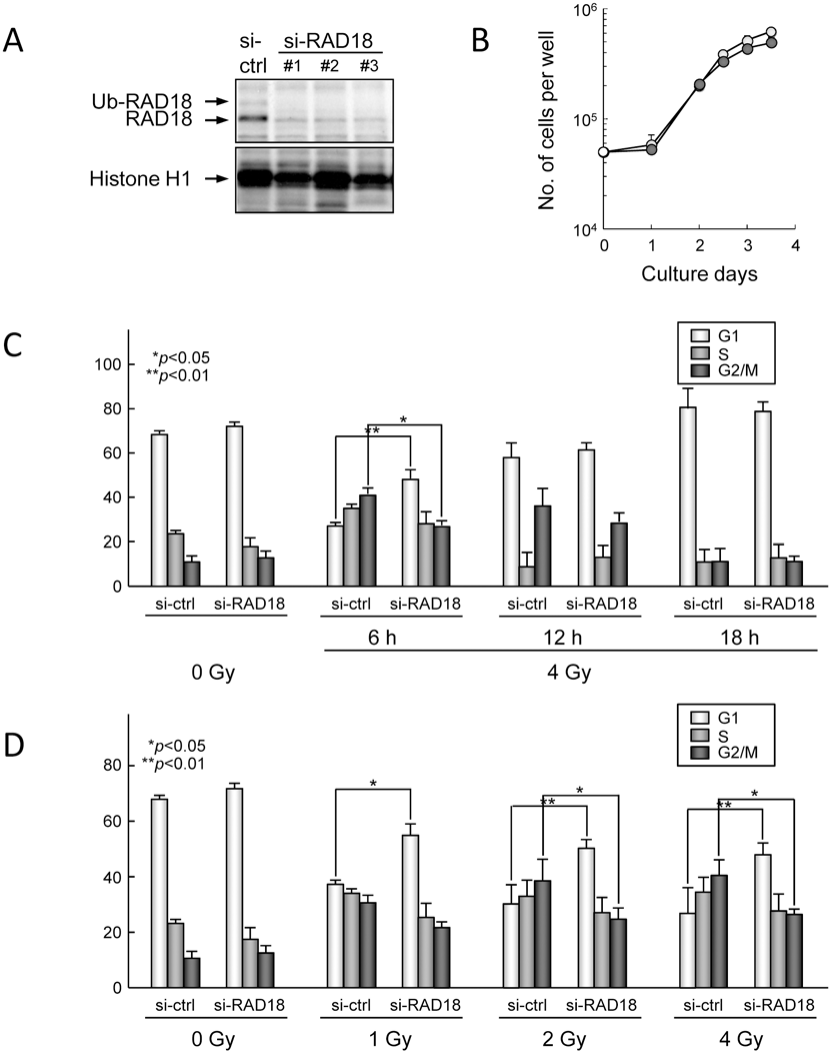
RAD18 is involved in IR-induced activation of the G2/M phase cell cycle checkpoint. (A) HT1080 human cancer cells were transfected with si-ctrl or si-RAD18. After 48 hrs, the expression levels of RAD18 in the insoluble fractions were determined by western blotting. (B) Cell proliferation was measured in si-ctrl and si-RAD18 treated cells. Each value represents the mean (+standard deviation) of the results from three independent experiments. (C) Cells were exposed to 4Gy IR, fixed at the indicated time points after irradiation, stained with propidium iodide (PI), and the cell cycle distributions were determined by flow cytometry. (D) Cells were exposed to 1, 2, and 4 Gy IR, and then fixed at 6 hrs after irradiation. Fixed cells were stained with PI and analyzed by flow cytometry.

To investigate roles of RAD18 in cell cycle checkpoint activation after IR exposure, we analyzed the distribution of the cell cycle using flow cytometry in HT1080 human cancer cell line treated with a non-targeted siRNA (si-ctrl.) or siRNA that targeted RAD18 (si-RAD18). Treatment with si-RAD18 depleted the expression of RAD18 and ubiquitylated RAD18 in these cells to less than 10% (Fig. 1A) but did not affect cell growth; growth rates were similar between cells treated with the si-ctrl (hereafter referred to as control cells) and those with si-RAD18 (hereafter referred to as RAD18-depleted cells) (Fig. 1B) [11]. RAD18-depletion also did not affect the cell cycle distribution under non-irradiated conditions (Fig. 1C). An arrest at the G2/M phase was observed in control cells at six and 12 hrs after IR exposure (4 Gy) and the percentage of cells arrested at the G2/M phase increased in an IR dose-dependent manner (Fig. 1C, D). In contrast, such an arrest of RAD18-depleted cells at the G2/M in response to IR exposure was less obvious (Fig. 1C, D). The percentage of S phase cells was similar between control and RAD18-depleted cells. Similarly, an arrest at the S phase induced by UV exposure was more evident in control cells than in RAD18-depleted cells (S1 Fig.).

### RAD18 depletion diminished IR-induced suppression of M phase entry

**Fig 2:**
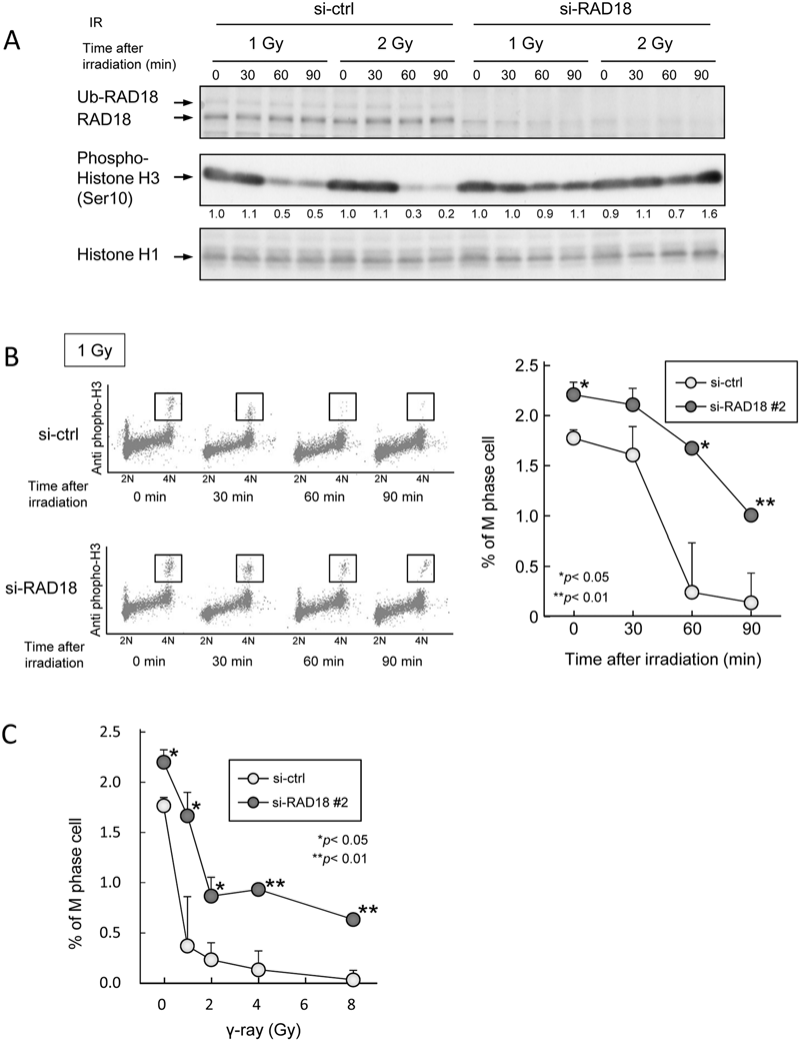
Depletion of RAD18 suppressed entry into the M phase from the G2 phase after IR exposure. (A) HT1080 cells transfected with si-ctrl or si-RAD18 were exposed to 1 or 2 Gy IR, and then lysed at the indicated time points, after irradiation. Samples were analyzed by western blotting with the indicated antibodies. (B) Cells were exposed to 1 Gy of IR, fixed with ethanol at the indicated time points after irradiation, and then immunostained with phospho-histone H3 and propidium iodide (PI). The percentage of G2/M phase cells was determined by flow cytometry. Each value represents the mean (+standard deviation) of the results from three independent experiments. (C) Cells were exposed to various doses of IR and then fixed with ethanol 60 min after irradiation. The fixed cells were immunostained with phosphor-histone H3 and PI. The percentage of G2/M phase cells was determined by flow cytometry. Each value represents the mean (+standard deviation) of the results from three independent experiments.

We further investigated roles of RAD18 on G2/M checkpoint activation by measuring the mitosis marker histone H3 phosphorylated at Ser10 (phospho-histone H3) to identify the M phase cells in HT1080 cell line. The percentage of phospho-histone H3-positive M phase cells was similar between non-irradiated control and RAD18-depleted cells (Fig. 2A, lane 1, 5, 9 and 13). However, phospho-histone H3 protein levels in the control cells decreased 60 min after IR exposure, suggesting G2/M checkpoint activation along with blockage of M-phase entry (Fig. 2A). In contrast, phospho-histone H3-positive M-phase cells remained to almost constant level in the RAD18-depleted cell population 60 min after IR exposure, suggesting inefficient G2/M checkpoint activation for blocking the M phase entry (Fig. 2A). The lack of G2/M checkpoint activation in response to IR exposure due to the depletion of RAD18 was also confirmed in three other human cancer cell lines (S2 Fig.).

M-phase cells were quantified with 4N DNA content and positive-staining for phospho-histone H3 using flow cytometry. Before IR exposure, the percentages of M phase cells were about 1.6% and 2.3% in the control and RAD18-depleted cell populations respectively (Fig. 2B, C). However, 60 min after 1 Gy exposure, M phase cells in the control decreased to 0.2% while 1.6% of RAD18-depleted cells were at the M phase. Decrement of M phase cells after irradiation somewhat depended on IR dose, and; the percentage of M phase cells was higher in RAD18-depleted cells compared to control cells at each dose level, indicating that RAD18 was involved in activating the G2/M checkpoint after exposure to IR.

### RAD18 mediated the DNA damage response pathway induced by IR

**Fig 3:**
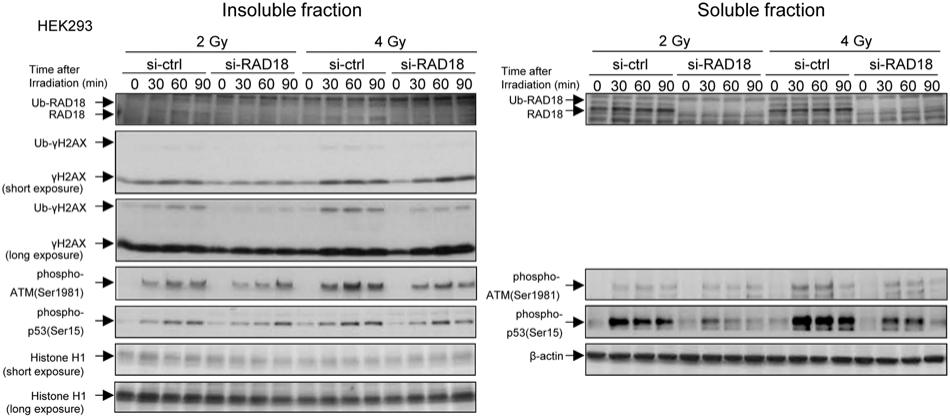
Depleting RAD18 suppressed the response to DNA damage induced by IR. HEK293 cells were transfected with si-ctrl or si-RAD18, irradiated with 2 or 4 Gy, and then lysed at the indicated time points after irradiation. Samples were analyzed by western blotting with the indicated antibodies.

To investigate effects of depleting RAD18 on the activation of the DNA damage signaling pathway in response to IR, we examined levels of ATM phosphorylated at Ser1981, H2AX phosphorylated at Ser139 (γH2AX) and p53 phosphorylated at Ser15, in RAD18-depleted cells compared to control cells in HEK293 cell line. Phosphorylated ATM, H2AX and p53 were evident in control cells 30 min after 2 Gy or 4 Gy irradiation, whereas phosphorylated ATM, H2AX and p53 levels were about 2~7 times lower than that in RAD18-depleted cells (Fig. 3). In addition, the level of ubiquitylated H2AX was about 7 times lower in the RAD18-depleted cells compared to the control cells (Fig. 3). These results suggest that Rad18 is involved in DNA damage signaling at DSB sites for activation of G2/M checkpoint following IR.

**Fig 4:**
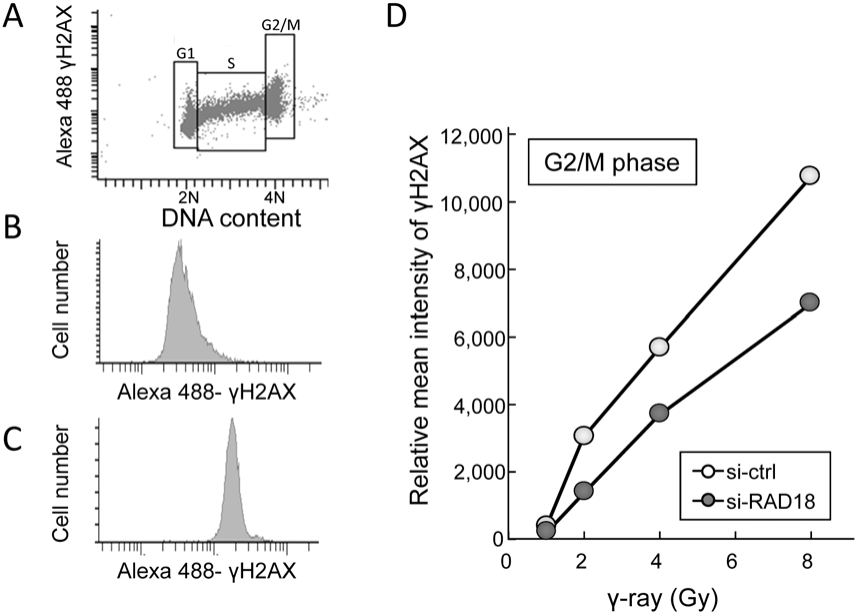
Depleting RAD18 reduced γH2AX levels at the G2/M phase in response to IR in HT1080 cells. (A) Gating on G1, S and G2/M cells. (B) γH2AX histograms of the gated G2/M phase population of non-irradiated cells. (C) γH2AX histograms of the gated G2/M phase population of cells irradiated with 8 Gy. (D) Dose dependent response of γH2AX fluorescence intensity in HT1080 cells. HT1080 cells transfected with si-ctrl or si-RAD18 were irradiated with 0,1,2,4 or 8 Gy IR and fixed with ethanol 60min after IR exposure. Fixed cells were immunostained with γH2AX and PI and analyzed by flow cytometry. Each value represents the mean (+standard deviation) of the results from three independent experiments.

Next, we aimed to confirm the role of RAD18 for DNA damage response at the G2/M phase. We measured levels of γH2AX as a marker for DNA damage signaling at the G2/M phase in the cells irradiated with 2 Gy of IR. Cell cycle stage was determined by DNA content (Fig. 4A). As shown in Fig. 4B and C, γH2AX levels were determined in G2/M phase cells with and without IR treatment. The levels of γH2AX increased linearly in both control and RAD18-depleted cells after irradiation in a dose-dependent manner (Fig. 4D). At the G2/M phase, levels of γH2AX fluorescence were lower in the RAD18-depleted cells compared to the control cells at any IR dose points we examined (Fig. 4D). Similarly, IR-induced γH2AX fluorescence was also lower at the G1 and S phases in RAD18-depleted cells than in the control cells (data not shown). These results indicate Rad18 deficiency causes less γH2AX induction in response to IR exposure.

**Fig 5:**
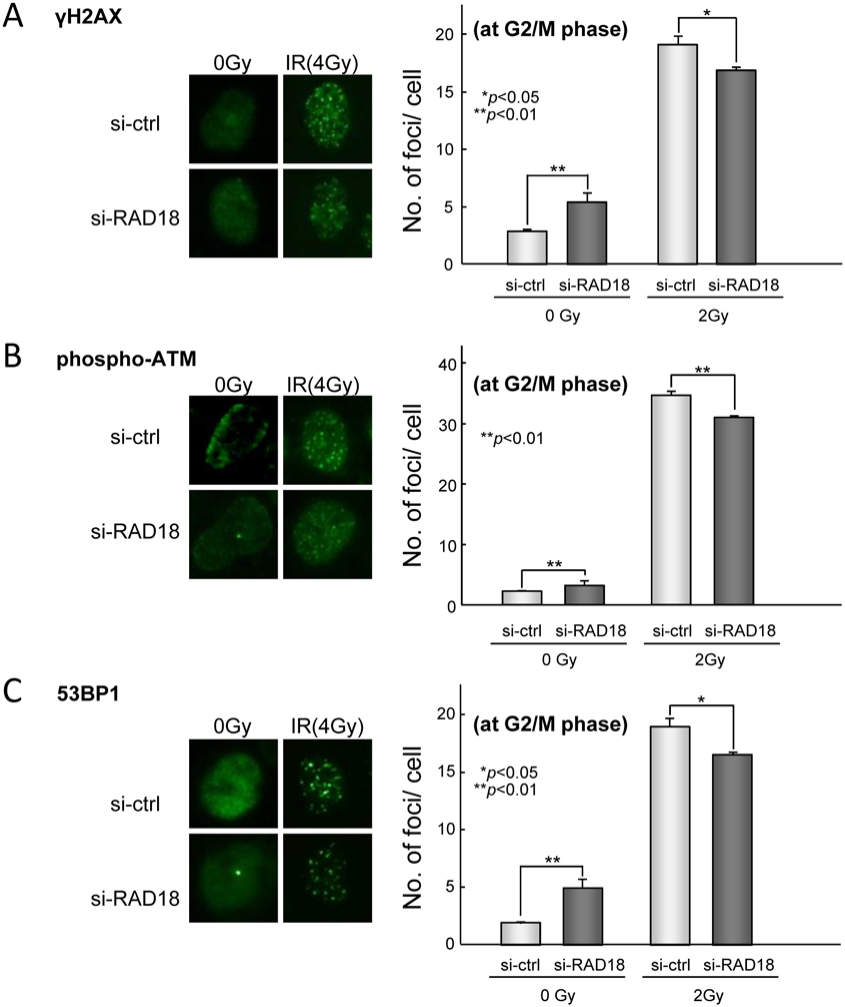
Depleting RAD18 suppressed foci formation at the G2/M phase by DNA damage signaling factors in response to IR. HEK293 cells transfected with si-ctrl or si-RAD18 were exposed to 2 Gy IR, labeled with EdU, and then fixed at 90 min after irradiation. The cells were co-immunostained with anti-Edu and anti-γH2AX, anti-phospho-ATM or anti-53BP1 antibodies. The G1, S, G2/M phase cells were distinguished using the IN Cell Analyzer. The number of foci per cell was determined using the image-analysis software of the IN Cell Developer. Each value represents the mean (+standard deviation) of the results from three independent experiments. Each value represents the mean (+standard deviation) of the results from three independent experiments. (A) γH2AX, (B) phosphor-ATM, (C) 53BP1.

We next examined effects of depleting RAD18 on recruitment of DNA repair proteins and formation of foci in G2/M phase cells using an IN Cell analyzer. Cell cycle stage was determined by DNA content and levels of 5-ethynyl-2’-deoxyuridine (EdU), with which S phase cells were specifically labeled. Under non-irradiated conditions, phosphorylated ATM (phospho-ATM), γH2AX and 53BP1 foci at the G2/M phase were more evident in RAD18-depleted cells than control cells (Fig. 5). In the cases of 2 Gy IR irradiation, however, fewer foci were induced in the RAD18-depleted cells compared to the control cells (Fig. 5). Furthermore, we examined focus formation with phosphorylated ATM, γH2AX and 53BP1 in RAD18-depleted cells at the G1 and S phase. Depletion of RAD18 had no effect on focus accumulation of these proteins at the S phase (S3 Fig.). The number of phosphorylated ATM-positive foci in RAD18-depleted cells was lower, but not significantly, compared to control cells at G1 phase (S3 Fig.). The numbers of γH2AX and 53BP1-labeled foci decreased in RAD18-depleted cells compared to control cells.

### The frequency of micronucleus formation in response to IR exposure was increased by RAD18 depletion both *in vitro* and *in vivo*

**Fig 6:**
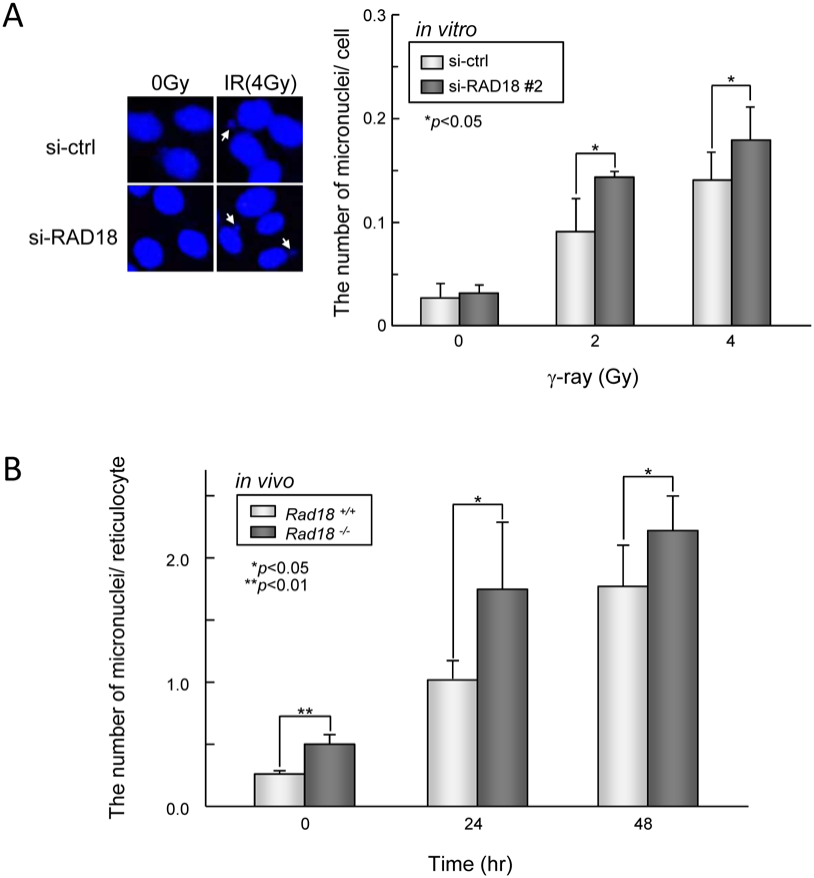
RAD18 is required for genomic stability after ionizing radiation exposure. (A) RAD18-depletion increased the frequency of micronuclei formation in response to IR *in vitro*. HT1080 cells transfected with si-ctrl or si-RAD18 were exposed to 2 or 4 Gy IR, fixed after 18 hrs, and then stained with Hoechst 33258 and SYTO RNASelect Green Fluorescent Cell Stain. The number of micronuclei per cell was determined using the IN Cell Analyzer. Each value represents the mean (+standard deviation) derived from three independent experiments. Each value represents the mean (+standard deviation) of the results from three independent experiments. (B) RAD18-deficiency increased the frequency of micronuclei formation in response to IR *in vivo*. *Rad18* ^*+/+*^ and *Rad18* ^-/-^ mice were irradiated with 1Gy IR. Peripheral blood was withdrawn from the tail vein at the time points indicated after irradiation, and micronucleated reticulocytes were labeled using a MicroFlow^PLUS^ kit. More than 20,000 reticulocytes from each mouse were analyzed by flow cytometry to determine the frequency of micronucleated reticulocytes. Each value represents the mean (+standard deviation) of the results for the individual mice.

To evaluate whether the depletion of RAD18 associated with the loss of the G2/M checkpoint leads to genome instability *in vitro*, we compared the frequency of micronucleus formation in control cells and RAD18-depleted cells exposed to IR. Depleting RAD18 increased micronucleus formation in HT1080 cells even without irradiation (Fig. 6A). Irradiation with 2 Gy or 4 Gy induced micronuclei in a dose-dependent manner in both the control and RAD18-depleted cells, but the number of RAD18-depleted cells that contained micronuclei was significantly higher compared to control cells at both doses (Fig. 6A). Next, to determine effects of Rad18 on genome stability *in vivo*, *Rad18* ^*+/+*^ and *Rad18* ^-/-^ mice were irradiated and percentages of micronucleated reticulocytes in the peripheral blood reticulocyte population were measured using flow cytometry. In non-irradiated mice, the percentage of micronucleated reticulocytes in *Rad18* ^*+/+*^ reticulocytes was 0.25%. This increased to 1.01% and 1.76% at 24 hrs and 48 hrs, respectively, after irradiating at 1 Gy (Fig. 6B). The percentage of micronucleated reticulocytes in non-irradiated *Rad18* ^-/-^ mice was significantly higher than those in reticulocytes from *Rad18* ^*+/+*^ mice, and this percentage increased after irradiation. Thus, these data suggest that Rad18 contributes to genome stability after IR exposure both *in vitro* and *in vivo*.

### Loss of RAD18 increased cell sensitivity to IR both *in vitro* and *in vivo*

**Fig 7:**
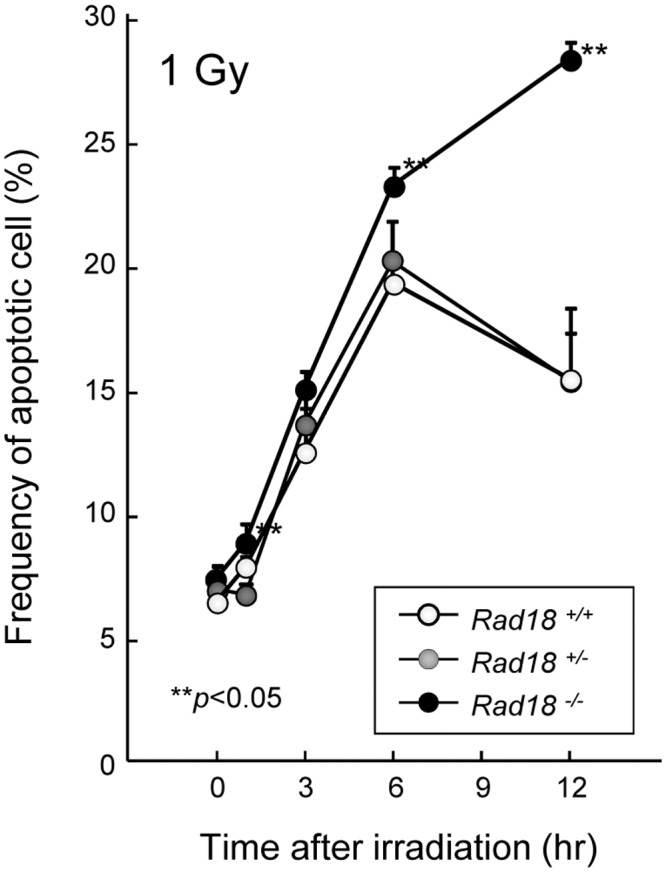
RAD18-deficiency increased apoptosis in murine thymocytes *in vivo*. *Rad18* ^*+/+*^, *Rad18* ^*+/-*^, and *Rad18* ^-/-^ mice were irradiated with 1Gy IR. Thymocytes were isolated at the time points indicated after irradiation. Apoptotic cell distributions in thymocytes were detected by using PE Annexin V Apoptosis Detection kit I and analyzed using flow cytometry. Each value represents the mean (+standard deviation) of the results from the individual mice.

To analyze effects of RAD18 on the sensitivity of cells to IR, survival rates were examined by colony assay in control and Rad18-depleted human HT1080 cancer cells after irradiation. RAD18-depleted cells were found to be moderately but significantly more sensitive to IR than the control cells (S4 Fig.). Next, we evaluated the frequency of apoptotic thymocytes induced by IR in *Rad18* ^*+/+*,^ *Rad18* ^*+/-*^ and *Rad18* ^-/-^ mice using an Annexin V binding assay and frequencies increased by 1-Gy IR irradiation and peaked at six hrs after the irradiation (Fig. 7). The frequency of apoptotic thymocytes was significantly higher in *Rad18* ^-/-^ mice compared to those in *Rad18* ^*+/+*^ and *Rad18* ^*+/-*^ mice at six hrs after exposure. At 12hrs after exposure, the level of apoptotic thymocytes had decreased in *Rad18* ^*+/+*^ and *Rad18* ^*+/-*^ mice whereas it was still increased in Rad18 ^-/-^ mice (Fig. 7). These results indicate that loss of RAD18 increases the sensitivity of cells to IR both *in vitro* and *in vivo*

## Discussion

RAD18 is involved in the PRR pathway via its ubiquitylation of PCNA but a recent study indicates that RAD18 plays a role in DSB repair that does not involve PCNA ubiquitylation.[21] It was reported that RAD18 promotes DSB repair during the G1 cell cycle phase and it is also involved in S phase-specific repair of DNA single-strand breaks.[9,13] However, functions of RAD18 in radioresponse of cells at the G2/M phase are well not understood. In this study, we first clarified the role of RAD18 in DNA damage signaling in cells at the G2/M phase after irradiation. Our data indicates that RAD18 is required for DNA damage signaling and activation of the G2/M checkpoint after exposure to IR, contributing to maintenance of genome integrity and cell survival.

### RAD18 functions as a molecular linker between DSB repair and checkpoint signaling in response to IR-induced DNA damage

IR causes a variety of DNA damages including DSBs, which are DNA lesions that are extremely toxic to cells. [19] To preserve genome integrity, cells use many systems including DNA damage repair and checkpoint pathways to respond to IR exposure. [20] Early in the response to IR, altered chromatin structures lead to the initial recruitment of ATM, followed by the phosphorylation of H2AX and the recruitment of additional factors such as MDC1, 53BP1 and RNF8. [22–26] These DNA damage signaling factors accumulate at the DSB sites that form after irradiation and are known as nuclear foci. [16] RAD18 is also recruited to the DSB sites and colocalizes with these factors (S5 Fig.). [9,17] It was reported that the recruitment of RAD18 depends on H2AX, MDC1 and RNF8 in the DNA damage signaling pathway after IR exposure but that recruitment of such DNA damage sensor as γH2AX, MDC1 and RNF8 occurs independently of RAD18 deficiency, [17] suggesting that RAD18 functions down stream of H2AX, MDC1 and RNF8. In addition, the localization of RAD18 to DSB sites is not affected in cells deficient for BRCA1, NBS1 or RAP80, suggesting that RAD18 functions up stream of these proteins in the IR-induced DNA damage signaling pathway. [17] Nevertheless, we showed that depletion of Rad18 reduces the intensity of IR-induced phospho-ATM, γH2AX and phosphorylated p53 (ser-15) following IR treatment of cells (Fig. 3), even though reportedly RAD18 is not necessary to recruit these factors.[17] RAD18 depletion decreased the number of phospho-ATM, γH2AX, 53BP1 and MDC1-labeled foci after ionizing radiation (Fig. 5 and S6 Fig.). We propose that RAD18 has a role in retention of these DNA damage-signaling factors at sites of DNA damage. Indeed, the initial recruitment and the subsequent retention of 53BP1 at DSB sites are mechanically distinct processes. [9,22,27,28] Although the initial recruitment of 53BP1 occurs independently of γH2AX, the stable retention of 53BP1 at DSB sites requires MDC1 and phosphorylated H2AX. Loss of 53BP1 or its functional failure leads to G2/M checkpoint defects and genome instability. [22,23,28] These results suggest that RAD18 may be involved in the efficient retention of DNA damage signaling factors.

It is well known that radiation can cause activation of the G2/M cell cycle checkpoint.[29] In this study, we report that the activation of the G2/M checkpoint following IR exposure was suppressed in cells depleted of RAD18. What kind of factor could function in cooperation with RAD18 during the G2/M phase to activate the G2/M checkpoint after IR exposure? Watanabe et al. reported that RAD18 interacts with 53BP1 and is recruited to DSBs in a 53BP1-dependent manner specifically during the G1 phase. [9] Given that the recruitment of RAD18 occurs independently of 53BP1 in the S and G2/M phases, they speculate that molecules other than 53BP1 are likely to associate with RAD18 in the S and G2/M phases. Recently, Inagaki et al. reported that RAD18 binds directly to ubiquitylated histone H2A and several other unknown ubiquitylated chromatin components via its zinc finger domain. [30] Histone H2A and H2AX that is located on damaged chromosomes is polyubiquitylated after IR exposure. [16] These polyubiquitin chains are known to assemble additional DNA damage signaling factors. [16,31,32] Our data indicates that following IR exposure, H2AX phosphorylation but also γH2AX ubiquitylation were suppressed by depleting cells of RAD18 [33]. We speculate that RAD18 interacts with the DNA damage signaling factors including histone H2A and H2AX, and retains them at the DSB sites in the G2/M phase. Further experimental investigation will be required to identify molecular mechanisms of the interactions between RAD18 and such DNA damage signaling factors at the G2/M phase after irradiation. We suggest that RAD18 functions as a molecular link between DNA damage repair and G2/M checkpoint signaling to efficiently promote the DNA damage response.

### RAD18 deficiency increases genome instability and sensitivity to IR

To analyze the effects of RAD18 on DNA repair capacity, we explored how the function of RAD18 was involved in genome stability and sensitivity to IR. The frequency of IR-induced micronucleus formation was increased by Rad18 deficiency both *in vitro* and *in vivo*, suggesting that RAD18 is required for genome stability. Indeed, neutral comet assay reveals that RAD18 depletion decreased DSB repair ability slightly but significantly (S1 Table). In accordance with a previous report,[34] we showed that RAD18-depleted human cancer cells have an increased sensitivity to IR. Furthermore, the frequency of IR-induced apoptosis was much higher in thymocytes from *Rad18* ^-/-^ mice compared those from *Rad18* ^*+/+*^ and *Rad18* ^*+/-*^ mice. We therefore hypothesize that IR-induced G2/M checkpoint activation is suppressed in proliferating thymocytes, such as immature CD8^+^ single positive cells and CD4^+^CD8^+^double positive cells of Rad18-/- mice. Loss of G2/M checkpoint due to RAD18 depletion would result in lower DNA repair capacity and apoptosis in the irradiated thymocytes from *Rad18* ^-/-^ mice. Future study is required to fully examine this hypothesis. We propose that Rad18 is one of the key factors that regulate genome stability and sensitivity to IR.

IR is one of the standard therapies for patients suffering from tumors. Recently, DNA damage signaling factors have received a great deal of attention as targets for molecular cancer therapies. In this study, we showed that Rad18 depletion modulates IR-induced genome instability and sensitivity *in vivo*. Furthermore, Xie et al. reported that Rad18 expression levels mediate resistance to IR in human glioma cells. [34] Therefore, we propose that RAD18 may be a suitable, novel target for cancer therapy, especially for treating recurrent, radioresistant tumors. Furthermore, synthetic lethal strategies are a new approach for cancer therapy to reduce off-target effects. Yamashita et al. has reported that double mutants of Rad18 and Rad54, a gene involved in HR, are synthetically lethal, whereas DT40 cells containing a single mutation in either gene can proliferate with nearly normal kinetics. [12] In addition, HCT116 cells deficient for Rad18 are lethal when they are also heterozygous for a mutation at the XRCC4 locus. [13] Further studies to clarify these interactions could provide novel approaches to synthetic lethal when strategies that involve RAD18 as a tool for new cancer therapies.

In summary, we have shown RAD18 plays a role in mediating the damage response signal to activate the G2/M checkpoint pathway and maintain genome integrity and cell survival after irradiation. RAD18-deficient mice should be a good tool that will increase our understanding about the role of RAD18 in radioresponses and IR-induced carcinogenesis *in vivo*.

## Supporting Information

S1 Fig RAD18 is involved in activation of the S phase cell cycle checkpoint induced by UV. (A) HT1080 cells transfected with si-ctrl or si-RAD18 were exposed to 2J/m^2^ UV, and then fixed at the time points indicated after UV treatment. Cells were stained with propidium iodide (PI) and the cell cycle distribution was analyzed using flow cytometry. (B) HT1080 Cells were exposed to 1, 2 or 4 J/m^2^ UV, and then fixed at 12 hrs after UV treatment. Fixed cells were stained with PI and analyzed using flow cytometry. (C) Cells were exposed to 1 or 2 J/m^2^ UV and lysed at the time points indicated after UV treatment. Samples prepared from the insoluble fractions were analyzed by western blotting with the indicated antibodies.
(DOCX)

S2 Fig Depleting RAD18 suppressed entry of G2 cells into the M phase after exposure to IR in other human cancer cell lines. H1299, HEK293 and HeLa human cancer cells transfected with si-ctrl or si-RAD18 were exposed to 2 Gy of IR and lysed at the time points indicated after irradiation. Samples prepared from the insoluble fractions were analyzed by western blotting with the indicated antibodies.
(DOCX)

S3 Fig Depleting RAD18 suppressed foci formation at G1 and S phase by DNA damage signaling factors in response to IR. HT1080 cells transfected with si-ctrl or si-RAD18 were exposed to 2 Gy IR, labeled with EdU, and then fixed 90 min after irradiation. The cells were co-immunostained with anti-BrdU and anti-γH2AX, anti-phospho-ATM or anti-53BP1 antibodies. The G1, S, G2/M phase cells were distinguished using the IN Cell Analyzer. The number of foci per cell was determined using the image-analysis software of the IN Cell Developer. Each value represents the mean (+standard deviation) of the results from three independent experiments.
(DOCX)

S4 Fig RAD18-depleted cells showed increased sensitivity to IR and UV. The sensitivity to IR (A) or UV (B) was analyzed using colony formation assays. HT1080 cells transfected with si-ctrl or si-RAD18 were exposed to increasing doses of IR or UV. Each value represents the mean (+standard deviation) of the results from three independent experiments.
(DOCX)

S5 Fig RAD18 colocalized with the IR-induced DNA damage signaling factors γH2AX, phospho-ATM and 53BP1 at the G1, S and G2/M phases. HT1080 cells were exposed to 4Gy IR, labeled with EdU, and then fixed at 60 min after irradiation. The cells were co-immunostained with anti-EdU and the indicated antibodies, then the G1, S, G2/M phase cells were distinguished using an IN Cell Analyzer.
(DOCX)

S6 Fig Depleting RAD18 suppressed foci formation at the G2/M phase by DNA damage signaling factors in response to IR. HT1080 cells transfected with si-ctrl or si-RAD18 were exposed to 2 Gy IR, labeled with EdU, and then fixed at 90 min after irradiation. The cells were co-immunostained with anti-BrdU and anti-NBS1 or anti- MDC1 antibodies. The G1, S, G2/M phase cells were distinguished using the IN Cell Analyzer. The number of foci per cell was determined using the image-analysis software of the IN Cell Developer. Each value represents the mean (+standard deviation) of the results from three independent experiments.
(DOCX)

S1 Table Neutral comet assay. (DOCX)
