# Supplementary material for: RAD18 Activates the G2/M Checkpoint through DNA Damage Signaling to Maintain Genome Integrity after Ionizing Radiation Exposure
Source: PLoS One. 2015 Feb 12;10(2):e0117845. doi: 10.1371/journal.pone.0117845 (PMC4326275; doi:10.1371/journal.pone.0117845)
Supplement: S1 Fig — (A) HT1080 cells transfected with si-ctrl or si-RAD18 were exposed to 2J/m2 UV, and then fixed at the time points indicated after UV treatment. Cells were stained with propidium iodide (PI) and the cell cycle distribution was analyzed using flow cytometry. (B) HT1080 Cells were exposed to 1, 2 or 4 J/m2 UV, and then fixed at 12 hrs after UV treatment. Fixed cells were stained with PI and analyzed using flow cytometry. (C) Cells were exposed to 1 or 2 J/m2 UV and lysed at the time points indicated after UV treatment. Samples prepared from the insoluble fractions were analyzed by western blotting with the indicated antibodies. (DOCX) [file pone.0117845.s001.docx]

**
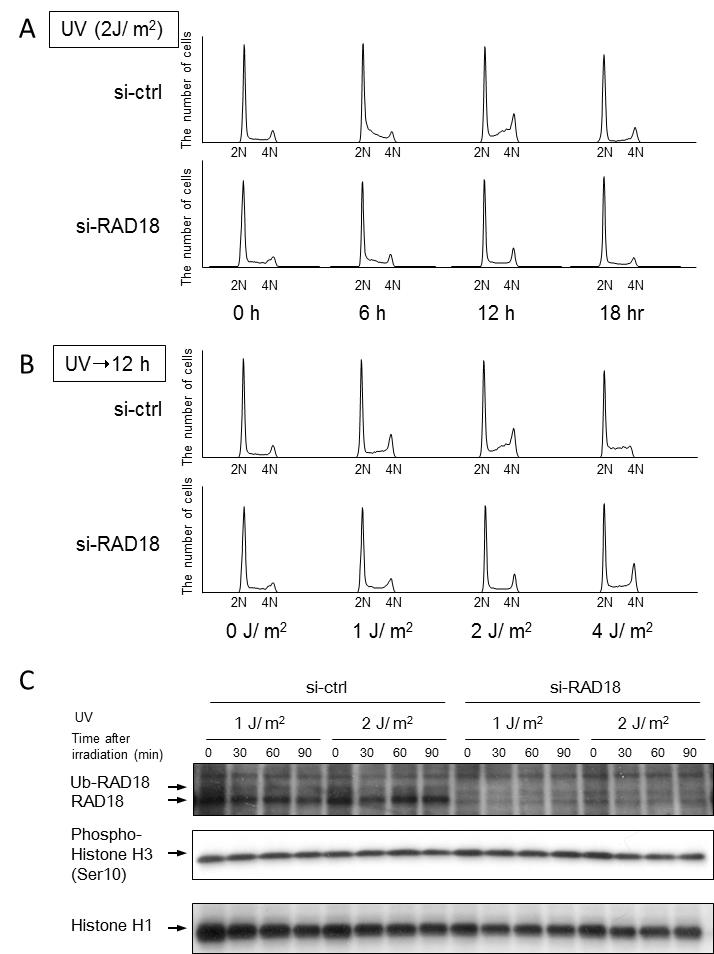
**

**Figure S1.** **RAD18 is involved in activation of the S phase cell cycle checkpoint induced by UV.** (A) HT1080 cells transfected with si-ctrl or si-RAD18 were exposed to 2J/m^2^ UV, and then fixed at the time points indicated after UV treatment. Cells were stained with propidium iodide (PI) and the cell cycle distribution was analyzed using flow cytometry. (B) HT1080 Cells were exposed to 1, 2 or 4 J/m^2^ UV, and then fixed at 12 hrs after UV treatment. Fixed cells were stained with PI and analyzed using flow cytometry. (C) Cells were exposed to 1 or 2 J/m^2^ UV and lysed at the time points indicated after UV treatment. Samples prepared from the insoluble fractions were analyzed by western blotting with the indicated antibodies.
