# Supplementary material for: RAD18 Activates the G2/M Checkpoint through DNA Damage Signaling to Maintain Genome Integrity after Ionizing Radiation Exposure
Source: PLoS One. 2015 Feb 12;10(2):e0117845. doi: 10.1371/journal.pone.0117845 (PMC4326275; doi:10.1371/journal.pone.0117845)
Supplement: S2 Fig — H1299, HEK293 and HeLa human cancer cells transfected with si-ctrl or si-RAD18 were exposed to 2 Gy of IR and lysed at the time points indicated after irradiation. Samples prepared from the insoluble fractions were analyzed by western blotting with the indicated antibodies. (DOCX) [file pone.0117845.s002.docx]

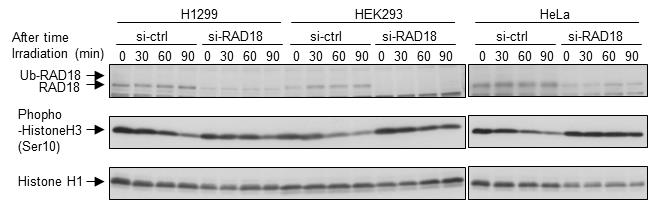


**Figure S2. Depleting RAD18 suppressed entry of G2 cells into the M phase after exposure to IR in other human cancer cell lines.** H1299, HEK293 and HeLa human cancer cells transfected with si-ctrl or si-RAD18 were exposed to 2 Gy of IR and lysed at the time points indicated after irradiation. Samples prepared from the insoluble fractions were analyzed by western blotting with the indicated antibodies.
