# Supplementary material for: RAD18 Activates the G2/M Checkpoint through DNA Damage Signaling to Maintain Genome Integrity after Ionizing Radiation Exposure
Source: PLoS One. 2015 Feb 12;10(2):e0117845. doi: 10.1371/journal.pone.0117845 (PMC4326275; doi:10.1371/journal.pone.0117845)
Supplement: S3 Fig — HT1080 cells transfected with si-ctrl or si-RAD18 were exposed to 2 Gy IR, labeled with EdU, and then fixed 90 min after irradiation. The cells were co-immunostained with anti-BrdU and anti-γH2AX, anti-phospho-ATM or anti-53BP1 antibodies. The G1, S, G2/M phase cells were distinguished using the IN Cell Analyzer. The number of foci per cell was determined using the image-analysis software of the IN Cell Developer. Each value represents the mean (+standard deviation) of the results from three independent experiments. (DOCX) [file pone.0117845.s003.docx]

**Figure S3. Depleting RAD18 suppressed foci formation at G1 and S phase by DNA damage signaling factors in response to IR.** HT1080 cells transfected with si-ctrl or si-RAD18 were exposed to 2 Gy IR, labeled with EdU, and then fixed 90 min after irradiation. The cells were co-immunostained with anti-BrdU and anti-γH2AX, anti-phospho-ATM or anti-53BP1 antibodies. The G1, S, G2/M phase cells were distinguished using the IN Cell Analyzer. The number of foci per cell was determined using the image-analysis software of the IN Cell Developer. Each value represents the mean (+standard deviation) of the results from three independent experiments.
