# Supplementary material for: RAD18 Activates the G2/M Checkpoint through DNA Damage Signaling to Maintain Genome Integrity after Ionizing Radiation Exposure
Source: PLoS One. 2015 Feb 12;10(2):e0117845. doi: 10.1371/journal.pone.0117845 (PMC4326275; doi:10.1371/journal.pone.0117845)
Supplement: S4 Fig — The sensitivity to IR (A) or UV (B) was analyzed using colony formation assays. HT1080 cells transfected with si-ctrl or si-RAD18 were exposed to increasing doses of IR or UV. Each value represents the mean (+standard deviation) of the results from three independent experiments. (DOCX) [file pone.0117845.s004.docx]

**Figure S4.** **RAD18-depleted cells showed increased sensitivity to IR and UV.** The sensitivity to IR (A) or UV (B) was analyzed using colony formation assays. HT1080 cells transfected with si-ctrl or si-RAD18 were exposed to increasing doses of IR or UV. Each value represents the mean (+standard deviation) of the results from three independent experiments.
