# Supplementary material for: RAD18 Activates the G2/M Checkpoint through DNA Damage Signaling to Maintain Genome Integrity after Ionizing Radiation Exposure
Source: PLoS One. 2015 Feb 12;10(2):e0117845. doi: 10.1371/journal.pone.0117845 (PMC4326275; doi:10.1371/journal.pone.0117845)
Supplement: S5 Fig — HT1080 cells were exposed to 4Gy IR, labeled with EdU, and then fixed at 60 min after irradiation. The cells were co-immunostained with anti-EdU and the indicated antibodies, then the G1, S, G2/M phase cells were distinguished using an IN Cell Analyzer. (DOCX) [file pone.0117845.s005.docx]

**Figure S5. RAD18 colocalized with the IR-induced DNA damage signaling factors γH2AX, phospho-ATM and 53BP1 at the G1, S and G2/M phases.** HT1080 cells were exposed to 4Gy IR, labeled with EdU, and then fixed at 60 min after irradiation. The cells were co-immunostained with anti-EdU and the indicated antibodies, then the G1, S, G2/M phase cells were distinguished using an IN Cell Analyzer.
