# Supplementary material for: RAD18 Activates the G2/M Checkpoint through DNA Damage Signaling to Maintain Genome Integrity after Ionizing Radiation Exposure
Source: PLoS One. 2015 Feb 12;10(2):e0117845. doi: 10.1371/journal.pone.0117845 (PMC4326275; doi:10.1371/journal.pone.0117845)
Supplement: S1 Table — (DOCX) [file pone.0117845.s007.docx]

Table S1. Neutral comet assay

| siRNA | γ-ray (Gy) | tail moment^a^ |
| --- | --- | --- |
| ctrl | 0 | 0.40 ± 0.47  * |
|  | 4 | 0.80 ± 0.53  *  * |
| RAD18 | 0 | 0.45 ± 0.41 |
|  | 4 | 1.18 ± 0.58 |

^a^Average number of tail moment. Values are means ±SD.

**p*<0.05
